# Dataset for damage detection retrieved from a monitored bridge pre and post verified damage

**DOI:** 10.1016/j.dib.2023.109729

**Published:** 2023-10-26

**Authors:** John Leander, Jacob Nyman, Raid Karoumi, Peter Rosengren, Gunnar Johansson

**Affiliations:** a KTH Royal Institute of Technology, Stockholm, 100 44, Sweden; b IoTBridge AB, Danderyd, 182 33, Sweden

**Keywords:** Structural health monitoring (SHM), Damage detection, Strain, Acceleration, Bascule bridge

## Abstract

The Vänersborg Bridge in southwest Sweden is a single-leaf bascule bridge carrying railway traffic over a canal. The load consists of passing commuter trains, occasional freight trains and leaf openings to allow ships to pass on the canal. The bridge constructed from 1914 to 1916 was built by riveted truss members in steel. Over the years, several assessments and maintenance actions have been performed to keep the bridge in service. During autumn 2021, a long-term monitoring campaign was initiated with the installation of sensors to register the load effect and possible changes in the behaviour. In March 2023, the cloud-based service employed detected an abrupt change of behaviour. An emergency inspection revealed a large crack in one of the truss members in the counter-weight part. The published dataset contains sensor data from 64 registered bridge openings, comprising accelerations, strains, inclinations, and weather conditions. Data from before the fracture, during, and after are provided. During the bridge opening events, the data was recorded continuously with a sampling rate of 200 Hz. The evidence of damage in a real case scenario makes the dataset valuable for testing and evaluation of data-driven routines for infrastructure surveillance.

Specifications Table

**Table:** 

Subject	Civil and Structural Engineering
Specific subject area	Structural health monitoring (SHM) and damage detection for bridges.
Type of data	Sensor data stored in CSV files.
How the data were acquired	Recorded by an on-site data acquisition (DAQ) system and transmitted to a cloud solution.
Data format	Raw
Description of data collection	Wired sensors for acceleration, strain, inclination, temperature, wind speed and direction connected to a DAQ with an integrated computer. The data was sampled at 200 Hz.
Data source location	• The Vänersborg Railway Bridge • Vänersborg • Sweden
Data accessibility	Repository name: KTH-Zenodo community URL: https://doi.org/10.5281/zenodo.8300495

## 1 Value of the Data

• The management of deteriorating transport infrastructure is a challenge for all developed countries. Many bridges have passed or are approaching their expected service life. Using sensors and cloud solutions to monitor the behaviour of bridges is emerging as a solution to remedy an increasing maintenance need. However, the value of monitoring is uncertain, and the ability to detect actual deterioration at early stages, before a failure occurs, is difficult to verify. The current dataset is one of few actual registered damages for a structure in service.
• The dataset will be of direct use to researchers and developers working with data-driven damage detection for large steel structures. From a long-term perspective, the data will benefit the development of future maintenance strategies for infrastructure owners.
• The sample of raw data captured the same bridge opening event multiple times over the monitoring duration. Classifying data before and after damage enables the development and verification of routines for novelty detection. With several entities measured, such as accelerations and strains, the data allows a critical review of appropriate methods and features.
• The bridge’s structural design is complex and deficiently described in the available drawings. It is infeasible to present a complete description of all the structural members of the bridge allowing structural modelling. The available data is judged appropriate for data-driven structural health monitoring (SHM) and damage detection methods.

## 2 Data Description

The dataset comprises 64 CSV files with raw data from the monitoring system on the bridge. More files can be made available upon request by IoTBridge AB. The files are named with the date and time for the first data point in the file. The system time for the DAQ was set to Coordinated Universal Time (UTC). Each file has one header line with variable names followed by numerical data.

Before sampling with 200 Hz, the analogue signals from the sensors were filtered with a Bessel lowpass filter and a threshold of 50  Hz.

### 2.1 Sensor data

The sensor data in the files is listed with one column per channel in the monitoring system. The order of the sensors in the files is listed in Table 1, with SG denoting strain gauges and A accelerometers. Columns 31 to 33 comes from a weather station which sampled the data in 4 Hz but was resampled in the DAQ to fit the general 200 Hz sampling. The data in the files are actual values already adjusted for gauge factors. The columns in Table 1 with a dash (-) for the sensor are channels in the DAQ without any sensor connected. The numerical values in the data files for these columns are irrelevant output from incomplete electrical circuits.

**Table 1:** Sensor order in the data files. SG denotes strain gauges, A accelerometers, and Inc the inclinometer.

Column	Header	Sensor	Unit	Column	Header	Sensor	Unit
1	-	Row count		20	ch_17	-	
2	ts	Date & time		21	ch_18	A1	m/s${}^{2}$
3	id	Index		22	ch_19	A2	m/s${}^{2}$
4	ch_1	SG1	µm/m	23	ch_20	A3	m/s${}^{2}$
5	ch_2	SG2	µm/m	24	ch_21	A4	m/s${}^{2}$
6	ch_3	SG3	µm/m	25	ch_22	A5	m/s${}^{2}$
7	ch_4	SG4	µm/m	26	ch_23	-	
8	ch_5	SG5	µm/m	27	ch_24	-	
9	ch_6	SG6	µm/m	28	ch_25	Inc	deg.
10	ch_7	SG7	µm/m	29	ch_26	-	
11	ch_8	SG8	µm/m	30	ch_27	-	
12	ch_9	SG9	µm/m	31	ch_28	Wind dir.	deg.
13	ch_10	SG10	µm/m	32	ch_29	Wind speed	m/s
14	ch_11	SG11	µm/m	33	ch_30	Air temp.	${}^{\circ }$C
15	ch_12	SG12	µm/m	34	fileid	File id.	
16	ch_13	SG13	µm/m	35	event	-	
17	ch_14	SG14	µm/m				
18	ch_15	SG15	µm/m				
19	ch_16	SG16	µm/m				

The data files are stored in ASCII format with a decimal comma (,) as the delimiter.

**Fig. 1:**
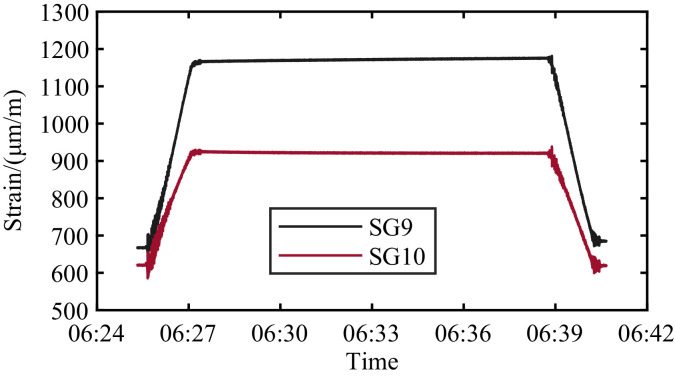
Raw data for the sensors SG9 and SG10 during a registered bridge opening Wednesday 8 March 2023.

**Fig. 2:**
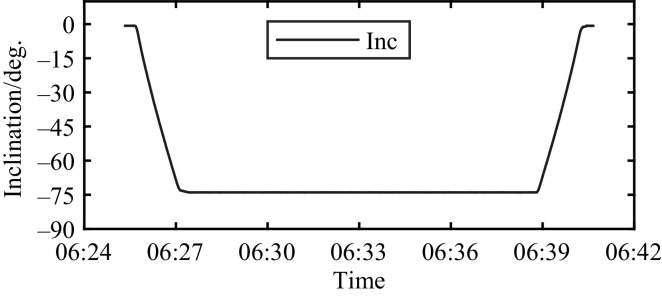
Raw data for the inclinometer (Inc) during a registered bridge opening Wednesday 8 March 2023.

Examples of the raw data are shown in Fig. 1 for two strain gauges and in Fig. 2 for the inclinometer. As shown for the strain gauges, they are not levelled at zero at the beginning of the load event. They have a level at resting condition that differs between gauges and varies with temperature. The strain gauges’ output should be levelled in the analysis to isolate the response of the loading events. The inclinometer data shown in Fig. 2 are absolute values reflecting the inclination of the leaf. The turning of the leaf upwards gives negative values.

### 2.2 Damage event

The cloud-based bridge monitoring service developed by IoTBridge AB detected an anomaly in the data starting with the opening event on 9 March 2023, at 23:45. For verification, the data has been reviewed thoroughly, and a change of behaviour pre- and post-event is evident. Moreover, the bridge manager ordered an emergency inspection and a large crack was discovered close to SG9 and SG10, for which the anomaly event was shown most clearly.

**Fig. 3:**
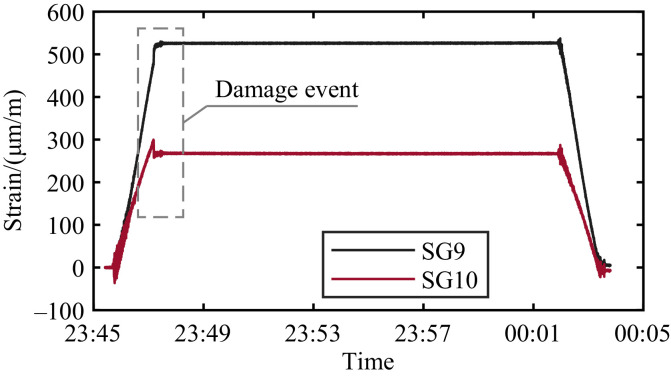
Data for the sensors SG9 and SG10 during the identified damage event Thursday, 9 March 2023. The time histories have been shifted to start at zero strain.

**Fig. 4:**
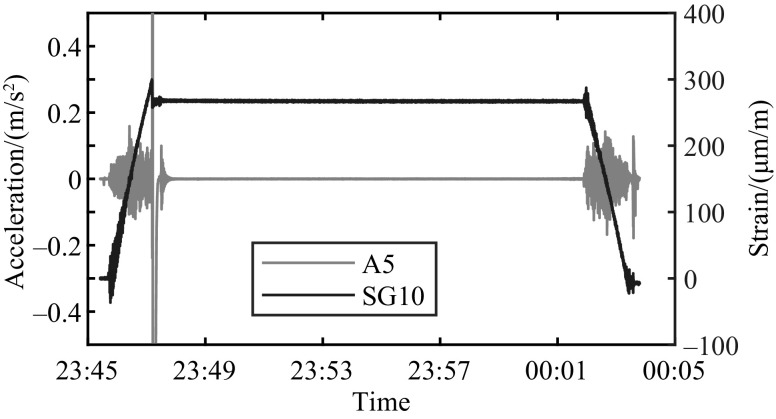
Data for the sensors A5 (left y-axis) and SG10 (right y-axis) during the identified damage event Thursday, 9 March 2023.

The actual damage event when a crack propagated in a member of the counterweight truss is registered in file 2023_03_09T23_45_25.csv and shown in Fig. 3 with measured strains during opening and closing. The time histories have been shifted to start at zero strain. The damage event is also traceable in the results for some of the accelerometers, e.g., A5, as shown in Fig. 4. The same instant a jump is registered in SG10, the measured acceleration jumps between extreme values.

## 3 Experimental Design, Materials and Methods

The monitored bridge, the instrumentation and the data collection procedure are described in the following subsections. More information about the bridge can be found in [2], and the monitoring setup is described in [3].

### 3.1 The bridge

**Fig. 5:**
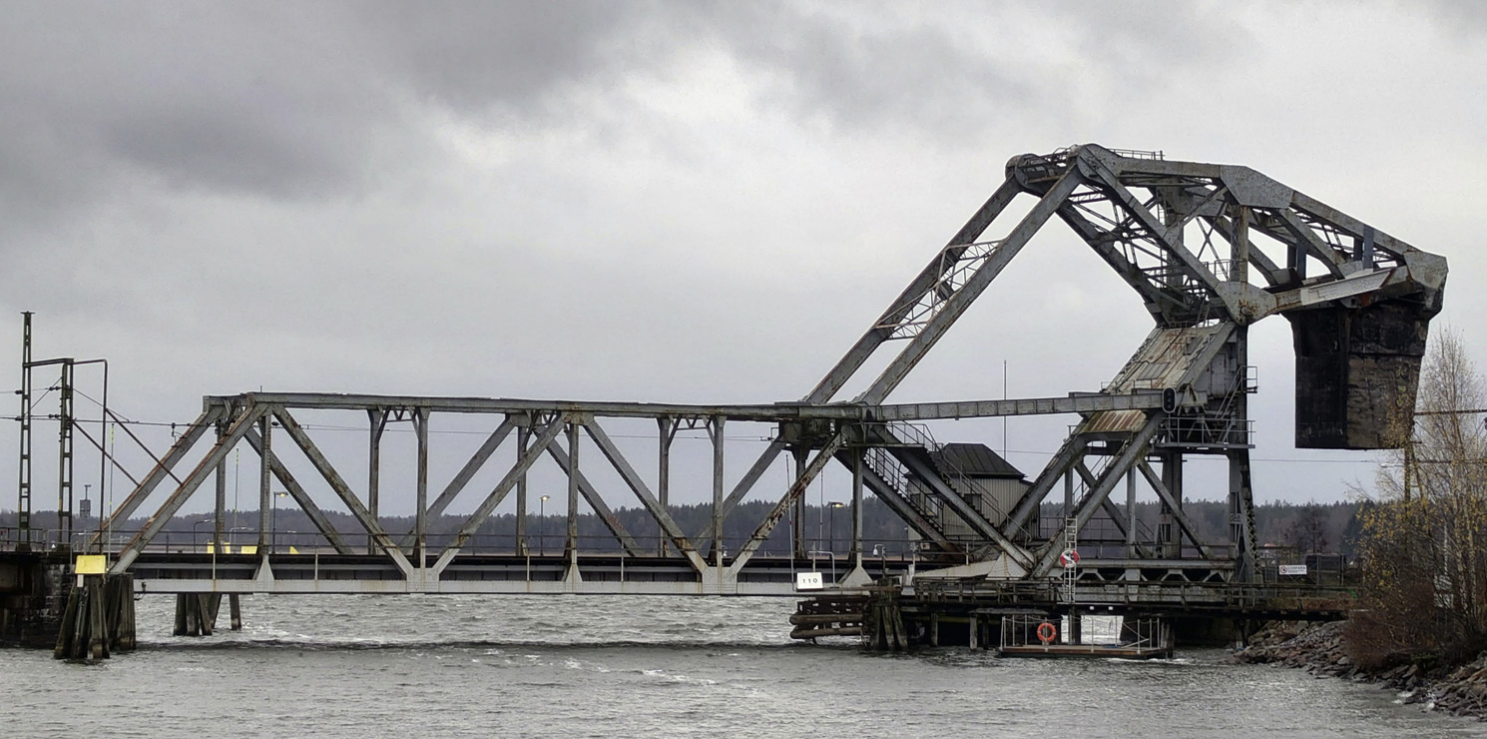
A photo of the Vänersborg Bridge.

The bridge in Vänersborg, Sweden, was built from 1914 to 1916 by the German company J. Gollnow und Sohn. It has the design of a heel trunnion bascule bridge often attributed to Joseph B. Strauss and sometimes called a Strauss bascule. The structural members are built up by riveted plates and angles. A photo of the bridge is shown in Fig. 5. The counterweight seen to the right in the figure is made of reinforced concrete. The structural steel is classified as St37, with a yield stress of 240 MPa and an elastic modulus of 210 GPa. A more detailed description of the bridge geometry can be found in [2].

### 3.2 Instrumentation

**Fig. 6:**
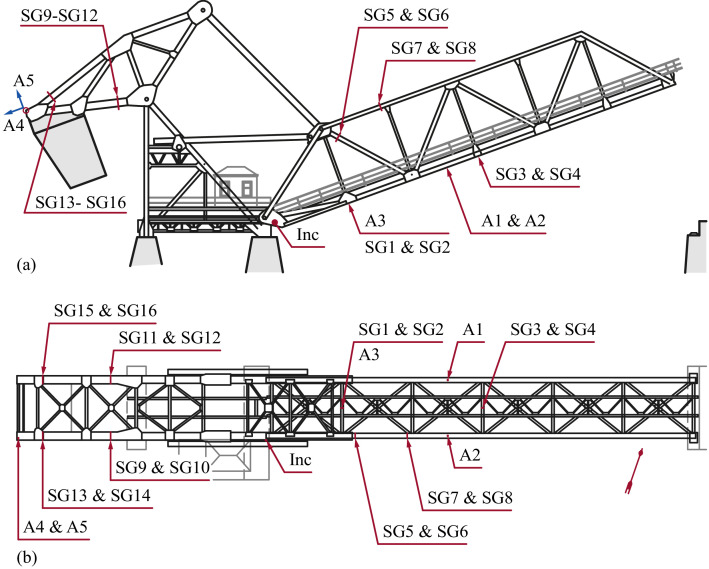
View of the bridge from (a) the side and (b) above, with the sensor locations.

The sensor locations are indicated in Fig. 6. The inclinometer (Inc) of type SST 141 from Vigor Technology was placed at the plate close to the main trunnion. From closed to open position, the inclinometer returns values from zero to $-90^{\circ }$. The bridge owner assigned an opening angle of a maximum of $\left(-\right)75^{\circ }$, but some exceedances were registered during monitoring.

The accelerometers A1 to A5 were of type 393A03 from PCB Piezotronics, with a frequency range of 0.5 to 2000 Hz and a sensitivity of 1 V/g. Accelerometer A4 measured in the horizontal direction when the bridge was in the closed position. All others measured vertically. During the opening event, the axis of operation turned with the bridge for all accelerometers. The blue arrows at the position of A4 and A5 in Fig. 6(a) indicate how the axes turned with the bridge.

**Fig. 7:**
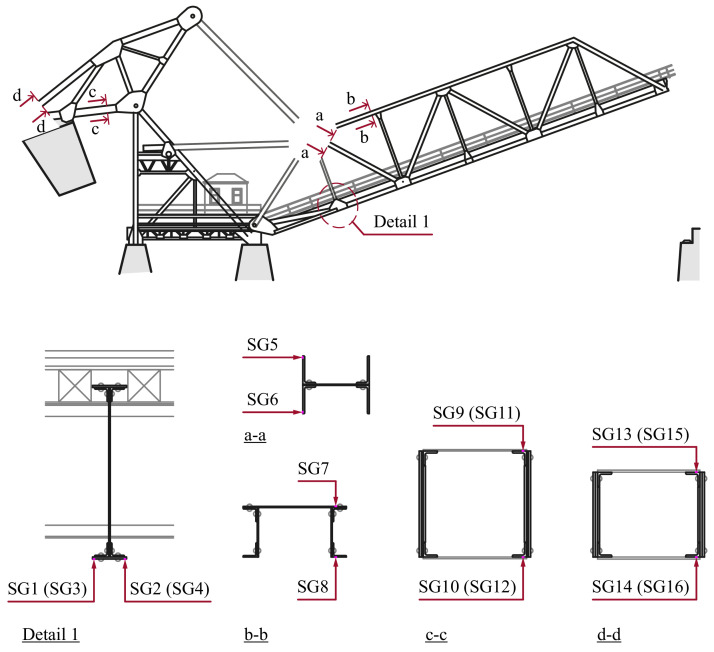
Cross-sections of the truss members where the strain gauges were mounted. The upper illustration shows where the sections are located. Detail 1 is a cross-section of the transversal beam between tha main trusses.

Weldable uniaxial strain gauges from HBM of type LS31HT-6/350VE were used for the strain measurements. They were mounted to the outer edges of the cross-sections. The positions on the members are indicated in Fig. 7. All strain gauges (SG1 to SG16) measured axial stresses along the members to which they were attached.

A weather station of type Vaisala Weather Transmitter WXT536 was installed at the bridge site. It measured pressure, air temperature, humidity, rain, wind speed and wind direction. It was connected to the main DAQ, but only wind direction, wind speed and air temperature were registered and synchronized.

### 3.3 Data collection and processing

The monitoring system was controlled at the site by a data acquisition (DAQ) unit from HBM of type CX22B-W. Amplifiers for the sensors were connected to this unit, with an MX1615B for the strain gauges and an MX840B for the accelerometers and the inclinometer. The data was sampled at 200 Hz after filtering with a lowpass Bessel filter with a cut-off frequency of 50 Hz.

The raw data files consisting of 10 min were stored on-site and transmitted to the cloud-based service provided by IoTBridge AB. Data processing is performed in the cloud. The service includes parsing, filtering, cleaning and storage in a database, building on the standard OGC SensorThings [1] for exchanging IoT data. The machine learning algorithms for damage detection implemented in the cloud issued an alarm for the anomaly caused by the actual damage. A detailed description of the software architecture implemented can be found in [3].

The dataset appended to this paper is the raw data from the DAQ on the bridge. However, the cloud service enabled the detection of the damage event and identified all files with bridge opening events provided in the dataset.

### 3.4 Damage event

**Fig. 8:**
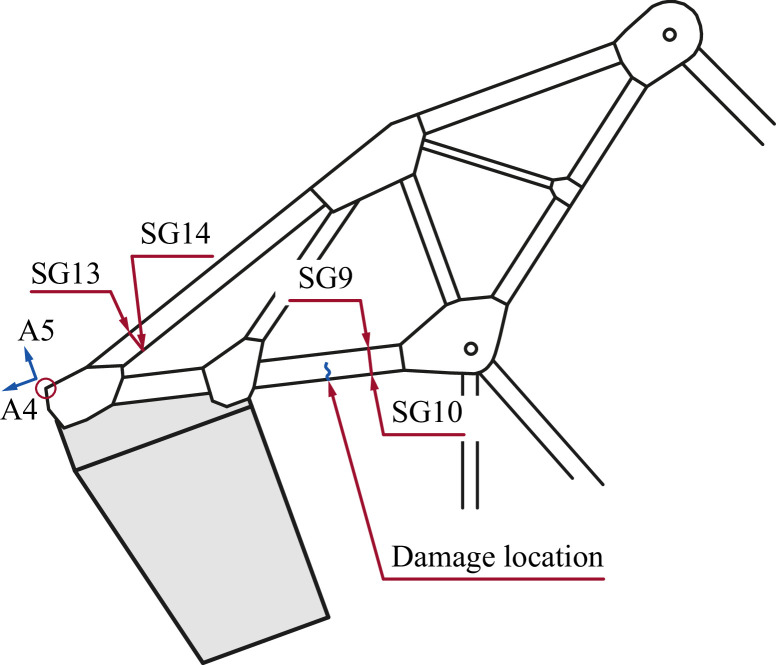
Position of the detected crack and the adjacent sensors.

**Fig. 9:**
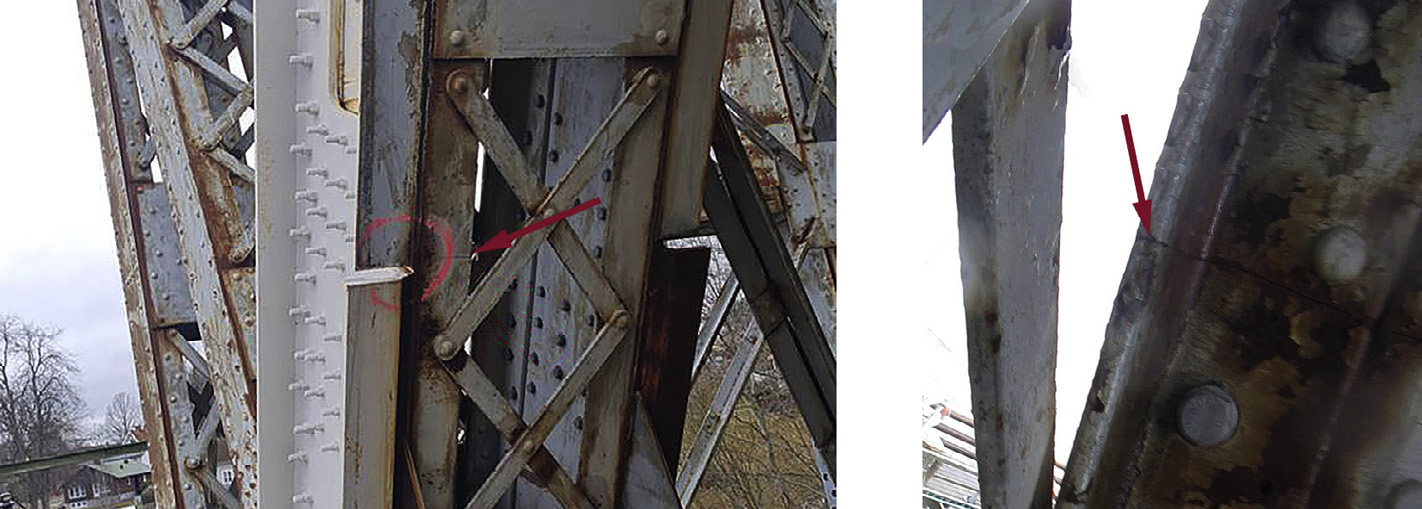
The picture to the left shows the bottom side of the member where the damage was observed. The right picture was taken from the inside of the cross-section showing the crack in the angle profile constituting the bottom flange. The arrows point at the crack.

The damage event was, by visual inspections, verified as a partial cracking of a truss member in the counterweight part. The bottom flange and a part of the web were affected. The crack location is indicated in Fig. 8 and images of the crack are shown in Fig. 9.

At the time of writing, the cracked member and the corresponding one on the other side have been strengthened, and the bridge is scheduled for replacement.

## Ethics Statements

The authors affirm that they did not involve any human subjects or animal experiments associated with this research. The authors also affirm that they did not involve any data collected from social media platforms.

## CRediT authorship contribution statement

**John Leander:** Conceptualization, Methodology, Writing – original draft. **Jacob Nyman:** Software, Data curation, Writing – review & editing. **Raid Karoumi:** Investigation, Resources, Writing – review & editing. **Peter Rosengren:** Data curation, Project administration, Writing – review & editing. **Gunnar Johansson:** Project administration, Funding acquisition.

## Declaration of Competing Interest

The authors declare that they have no known competing financial interests or personal relationships that could have appeared to influence the work reported in this paper.

## Data Availability

Dataset from structural health monitoring of a steel bridge in Sweden (Original data) (Zenodo).
